# *COMT* Genetic Variants and BDNF Level Associations with Cannabinoid Plasma Exposure: A Preliminary Study

**DOI:** 10.3390/jox15030066

**Published:** 2025-05-07

**Authors:** Alessandra Manca, Cristina Valz, Francesco Chiara, Alice Palermiti, Jacopo Mula, Sara Soloperto, Miriam Antonucci, Amedeo De Nicolò, Nicola Luxardo, Daniele Imperiale, Flavio Vischia, David De Cori, Jessica Cusato, Antonio D’Avolio

**Affiliations:** 1 Laboratory of Clinical Pharmacology and Pharmacogenetics, Department of Medical Sciences, University of Turin, Amedeo di Savoia Hospital, 10149 Turin, Italy; 2 SC Terapia del Dolore, ASL Città di Torino, 10144 Turin, Italynicola.luxardo@aslcittaditorino.it (N.L.); 3 Laboratory of Clinical Pharmacology San Luigi A.O.U., Department of Clinical and Biological Sciences, University of Turin, 10043 Turin, Italy; 4 SCDU Infectious Diseases, Amedeo di Savoia Hospital, ASL Città di Torino, 10149 Turin, Italy; 5 Neurology Unit, Maria Vittoria Hospital, ASL Città di Torino, 10144 Turin, Italy; 6 Psychiatric Unit West, Department of Mental Health, 10149 Turin, Italy

**Keywords:** THC, genetics, cannabinoids, pain, BDNF

## Abstract

*Cannabis sativa* L. shows potent anti-inflammatory activity, resulting in an interesting pharmacological option for pain management. The aim of the study was to evaluate the association between pharmacogenetics, neurological and inflammatory biomarkers, and cannabinoid plasma exposure in patients treated with cannabis. A total of 58 patients with a diagnosis of neuropathic and chronic pain treated with medical cannabis were analyzed. Cannabis was administered as a decoction (n = 47) and as inhaled cannabis (n = 11): 30 patients were treated with cannabis with high THC, while 28 patients were treated with cannabis with reduced THC (plus CBD). Cannabinoid plasma concentrations were obtained with UHPLC-MS/MS. Allelic discrimination was assessed by real-time PCR. Inflammation biomarkers (e.g., interleukin-10) were analyzed by ELISA, neurofilaments light chain (NFL), and brain-derived neurotrophic factor (BDNF) by Single Molecule Array. A statistically significant difference in IL-10 (*p* = 0.009) and BDNF (*p* = 0.004) levels was observed comparing patients treated with decoction and inhaled cannabis. BDNF and NFL results correlated with cannabinoid concentrations. Concerning genetics, the *COMT* 680 T>C genetic variant influences cannabinoid plasma levels, including Δ9-THC (*p* = 0.017). Conclusions: This study shows a possible impact of some genetic variants on cannabinoid plasma exposure, other than a possible role of medical cannabis on inflammation-related and neuronal impairment factor levels. Further studies in larger cohorts are required.

## 1. Introduction

*Cannabis sativa* L. use has been reported in about 147 million people worldwide [1]. It is one of the most ancient cultivated plants due to its adaptability to a wide range of habitats and its multiple uses, including food, fiber, and a drug plant [2].

The first evidence of its utilization in humans dates back to about 10,000 years ago: cannabis fruit and seed fossils have been found in the Okinoshima archeological site of the Mesolithic Age (Boso Peninsula, central Japan) [3].

In Italy, the authorization, cultivation, import, export, and distribution of cannabis are regulated by the Ministerial Decree of 9 November 2015 [4].

The main therapeutic indication for the use of medical cannabis (MC) is analgesia in pathologies with spasticity associated with pain (such as in multiple sclerosis) and in chronic pain, in particular in neurogenic pain.

MC contains a large variety of chemical compounds, including the psychoactive cannabinoid delta-9-tetrahydrocannabinol (∆9-THC) and the non-psychoactive cannabidiol (CBD). These cannabinoids interact with the endocannabinoid system which plays an important role in several physiological processes such as nervous functions, neurogenesis, neuroprotection, depression, memory, cognition, and painful sensation [5,6,7].

The endocannabinoid system resulted in an interesting pharmacological target for many diseases, including pain and neurodegenerative conditions [8,9,10].

Cannabinoid-receptor type 1 (CB1) is present in the central nervous system (in the neocortex, cerebellum, and limbic system) and in the peripheral nervous system. Cannabinoid-receptor type 2 (CB2) has been identified in lymphocytes, macrophages, and mast cells in the immune system and in microglia cells and astrocytes in the central nervous system [11]. CB1 activation mediates drug and natural rewards, such as sexual activity, social interaction, and food consumption [12]. CB2 receptor agonists exhibit antinociceptive activity in models of inflammatory and nociceptive pain, modulating the immune responses and reducing the release of pro-inflammatory cytokines [13]. These effects may be further enhanced through interactions with other systems, such as activation of the peroxisome proliferator-activated receptor α (PPAR-α), and modulation of transient receptor potential vanilloid type 1 (TRPV1) and α2-adrenoceptor pathways [14,15,16].

Furthermore, cannabinoids show activity against different types of pain: thermal and noxious, cancer, postoperative and that related to spinal cord and traumatic nerve injury and toxic insults [17,18]. These molecules have also potent anti-inflammatory activity: over-inflammation is present in many pathologies, such as cancer, asthma, rheumatoid arthritis, multiple sclerosis, hepatitis, colitis, and dermatologic diseases [7]. ∆9-THC and CBD showed anti-inflammatory and immune-suppressive properties interacting with CB1 and CB2 receptors [19]. In this context, in a study in a murine model [20], the GPR55 receptor modulates proinflammatory cytokines (such as interleukin (IL)-4, IL-10, interferon gamma), reducing hyperalgesia.

Cannabinoids are able to downregulate the production of cytokines and chemokines, suppressing inflammatory responses [19]: as an example, CBD could modulate inflammation influencing the release of some pro-inflammatory cytokines, such as tumor necrosis factor α (TNF-α) [21]. No data on possible associations between cannabinoid plasma exposure and inflammation-related biomarkers are present in the literature.

Furthermore, the role of CBD in reducing depression by increasing brain-derived neurotropic factor (BDNF) has also been suggested [22]. BDNF is a neurotrophin involved in the survival, activity, and growth of neurons [23]. It has been recognized as an important pain modulator, regulating central and peripheral synaptic plasticity. It is also involved in neuropathic and inflammatory pain due to its role in sensory neurotransmission in spinal and supraspinal level nociceptive pathways. In a study, elderly individuals with higher peripheral BDNF levels showed reduced chances of developing Alzheimer’s disease [24]. BDNF exposure resulted in changes in other neurodegenerative and mental health disorders [25]. Reduced BDNF levels may indicate a lack of trophic support, potentially contributing to neuronal degeneration [26]. For these reasons, BDNF has been proposed as a biomarker of neuroprotection [27,28].

In the context of tailored medicine, pharmacogenomics is a crucial tool for personalizing pain treatment with MC [29]. According to the literature, several genes encoding enzymes (such as cytochromes P450, CYP) are involved in the cannabinoid biotransformation [29,30], impacting the absorption, distribution, metabolism and elimination of these molecules, consequently affecting efficacy and adverse effect risk. THC is first metabolized in the liver by CYPs into 11-Hydroxy-Δ9-tetrahydrocannabinol (11-hydroxy-THC) and then oxidized into 11-Nor-9-carboxy-Δ9-tetrahydrocannabinol (THC-COOH), which may be glucuronidated to 11-nor-9-carboxy-THC glucuronide. The main hepatic enzymes involved in THC metabolism are CYP2C19, CYP2C9, and CYP3A4. Also, extra-hepatic tissues express CYPs, such as the brain and small intestine, and have a role in cannabinoid metabolism [31]. CBD is a substrate of CYP2C19, CYP1A1, CYP3A4, CYP1A2, CYP2C9, and CYP2D6, and it is mainly hydroxylated into 7-hydroxy cannabidiol (7-OH-CBD) [32].

In addition, the *ABCB1* gene encodes the P-glycoprotein, which is an efflux pump potentially affecting the distribution and bioavailability of MC metabolites [30,33].

Few data are available in this context; thus, the aim of the present preliminary study was to investigate inflammation-related and neurological marker levels in individuals treated with MC.

In addition, the impact of pharmacogenetics was evaluated: variants of genes encoding cytochromes and transporters involved in cannabinoid metabolism and transport were investigated.

## 2. Materials and Methods

### 2.1. Characteristics of Enrolled Patients

Patients with neuropathic and chronic pain treated with MC were enrolled at the “SC Terapia del Dolore—ASL Città di Torino” at the “Oftalmico” hospital (Turin, Italy). Nine patients were excluded from the statistical analysis due to incorrect MC decoction intake (e.g., skipped doses).

The study was performed in compliance with the Declaration of Helsinki and local review board regulations; all patients gave written informed consent, according to the local ethics committee standards (“Cannabis terapeutica nei pazienti affetti da dolore neuropatico: studio osservazionale”, approved by Ethical Committee “A.O.U. CITTA’ DELLA SALUTE E DELLA SCIENZA DI TORINO—A.O. ORDINE MAURIZIANO DI TORINO—A.S.L. CITTÀ DI TORINO”, n° 0131170 del 25 November 2022).

Inclusion criteria were MC treatment for at least 15 days; exclusion criteria included age under 18 years.

In this study, 47 individuals were treated with oral MC (decoction), while 11 patients were treated with inhaled MC.

For MC decoction preparation, the following procedure was used: 100 mL of cold water for every 100 mg of cannabis was used. It was recommended to use 100 mL of water. Heat to boiling and then let it simmer, covered, for 15 min (not exceeding 30 min total boiling time). The decoction should cool for about 15 min before filtering and then freshly consumed.

Inhalation as a route of administration was chosen by patients, in agreement with the clinician. Indeed, if oral administration (decoction) did not produce the expected pharmacological effect, or when the physician accounted it appropriate, inhalation was used as an alternative method of administration with the use of a personal vaporizer with filtered hot air.

The correct dose for each patient was determined by the clinician, based on the type of disease and pain severity: MC has been used to induce analgesia in patients resistant to conventional treatments (e.g., opioids). All the patients were naïve for MC administration.

Dried cannabis flowers for decoction preparation or for vaporization were provided by the hospital pharmacy at the institution, as a clinical routine. The frequency of the administration was once daily.

Patients were treated with cannabis with high THC, while 28 patients with low-THC cannabis formulations containing CBD.

Following dried flower tops of different varieties of *Cannabis* were used:

- Bedrocan^®^ (THC level standardized at 19% and with a CBD level < 1%);
- Bediol^®^ (THC and CBD levels standardized at concentrations of 6.5% and 8%, respectively);
- FM2^®^ (THC and CBD levels standardized at concentrations of 5–8% and 7.5–12%, respectively);
- Pedanios^®^ 22/1 (THC and CBD levels standardized at concentrations of 22% and <1%, respectively).

Bediol^®^ and Bedrocan^®^ (Bedrocan International BV, Veendam, The Netherlands) were the main varieties used; when they were not available, FM2^®^ (Military Chemical and Pharmaceutical Institution of Florence, Florence, Italy) and Pedanios^®^ (AURORA *Cannabis* Enterprises Inc., Edmonton, AB, Canada) were administered.

### 2.2. Pharmacokinetic Analyses

Cannabinoid metabolites were quantified in plasma after reaching the steady state (at least 15 days after starting therapy): plasma was obtained from blood withdrawals in EDTA tubes immediately before the dose administration of the day (C_trough_). Whole blood was centrifuged at 1400× *g* for 10 min at 4 °C, in order to obtain plasma samples which were stored at −80 °C until the analysis. Cannabinoid plasma levels were evaluated using a previously published fully validated method [34]. The whole chromatographic run was completed in 10.0 min and an optimal chromatographic separation between all analytes was obtained. Considered analytes were as follows: Δ9-THC (Δ9-tetrahydrocannabinol), 11-OH-THC (11-Hydroxy-Δ9-tetrahydrocannabinol), COOH-THC, COOH-THC-glucuronide, CBD, 7-OH-CBD, tetrahydrocannabinolic acid (THCA), and cannabidiolic acid (CBDA).

### 2.3. Genetic Polymorphism Analyses

Whole blood was collected in EDTA tubes: DNA was extracted using the QIAamp DNA mini kit (Qiagen, Valencia, CA, USA). These kits contain columns allowing DNA purification starting from 200 µL of whole blood.

Allelic discrimination was performed using RT-PCR (BIORAD, Milan, Italy).

The following allelic variants were analyzed: *ABCB1* 3435 C>T (rs1045642), *ABCB1* 2677 G>A (rs2032582), *ABCB1* 1236 C>T (rs1128503), *ABCG2 421* C>A (rs2231142), *ABCG2* 1194+928 T>C (rs13120400), *CYP2D6* 4180 C>G (rs1135840), *CYP1A1* 7294 C>A (rs2606345), *CYP1A2* 890 C>T (rs2470890), CYP2C19*2 G>A (rs4244285), *CYP1A2* 32035 A>C (rs762551), *BSEP* T>C (rs2287622), CYP3A4*1B G>A (rs2740574), CYP2C9*3 1075 A>C, CYP2C9*2 430 C>T, *COMT* 680 T>C (rs4680), *GHC1* 841 T>C (rs841), *OPRM1* 971 T>C (rs1799971), *TRPV1* 080 G>A (rs8065080).

These SNPs were selected because the corresponding genes encode for enzymes, transporters, and receptors involved in the metabolism, transport, and activity of cannabinoids. They were selected based on their allelic frequency, which is commonly represented in the Caucasian population.

### 2.4. ELISA Tests

In this study, the BT LAB kit direct method (Bioassay Technology Laboratory, Birmingham, UK) was used to quantify inflammation-related biomarkers in plasma. IL-6, IL-10, and TNF-α plasma levels were analyzed.

### 2.5. SiMoA^®^ Tests

Plasma specimens were analyzed using Single Molecule Array (SiMoA^®^ SR-X, Quanterix^®^ Billerica, MA, USA) for markers of neuronal damage, signaling, and plasticity: NFL and BDNF were assessed.

### 2.6. Statistical Analyses

The Shapiro–Wilk test was used to assess the normality of all continuous variables. The correspondence of each factor, normal or non-normal, was further evaluated using the Kolmogorov–Smirnov test. Non-normally distributed variables were described using median and interquartile range (IQR), while categorical variables were reported as counts and percentages. Kruskal–Wallis and Mann–Whitney tests were used to identify differences in continuous variables, with two-sided *p*-values < 0.05 considered statistically significant. Finally, the predictive power of the variables under investigation was assessed using univariate (*p* < 0.05) and multivariate (*p* < 0.05) linear regression analysis.

All statistical analyses were performed using IBM SPSS Statistics software, version 28.0 for Windows (Chicago, IL, USA).

## 3. Results

### 3.1. Characteristics of Enrolled Patients

In this study, 67 patients were enrolled at the “SC Terapia del Dolore—ASL Città di Torino” at the “Oftalmico” hospital (Turin, Italy); 9 patients were excluded from the statistical analysis due to incorrect MC decoction intake (e.g., missing doses).

Patients were affected by neuropathic and chronic pain caused by various conditions: 46.6% of patients had fibromyalgia, 8.6% had headaches, 6.9% were oncologic patients, and 65.5% were polytraumatized individuals.

Patient characteristics are reported in Table 1: 47 patients were treated with MC decoction, and 11 were treated with inhaled MC; no patient received both routes of administration. Additionally, 51.7% (n = 30) of patients were treated with MC with high THC content, while 48.3% (n = 28) received MC with lower THC and standardized CBD concentrations (6.5% and 8%, respectively).

The most common dose range was 0–250 mg of cannabis per day for patients treated with both oral and inhaled MC. All dosages are reported in Table 2.

Cannabinoid plasma levels (ng/mL) are reported in Table 3 and Table 4.

Most patients were on polypharmacy, considering their various diagnoses: 20 patients were treated with antidepressants (39.2%), 16 with anti-inflammatory drugs (31.4%), 21 with opioids (41.2%), 16 with anticonvulsants (31.4%), 15 with cardiovascular drugs (29.4%), 9 with vitamin D supplementation (17.6%), 17 with anti-anxiety medications (33.3%), and 26 with other drugs (89.7%), as reported in Supplementary Table S1.

### 3.2. Biomarker Concentrations

TNF-α, IL-6, IL-10, BDNF, and NFL concentrations were quantified in plasma, and their median concentrations are reported in Table 5.

**Figure 1:**
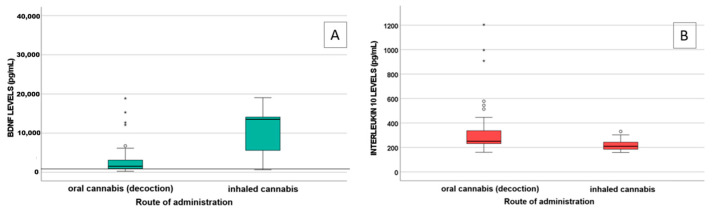
Influence of the route of administration (oral and inhaled cannabis) on BDNF levels (Panel (**A**), pg/mL) (*p* = 0.004) and on interleukin-10 levels (panel (**B**), pg/mL) (*p* = 0.009). Outliers are represented by little circles, and extreme outliers are represented by little stars, the line in A represents BDNF levels in healthy subjects.

A statistically significant difference in IL-10 (*p* = 0.009) and BDNF (*p* = 0.004) levels was observed between patients treated with decoction (n = 47) and those treated with inhaled MC (n = 11), as shown in Figure 1.

NFL and BDNF were found to be correlated with cannabinoid levels: specifically, NFL with THCA levels (*p* < 0.001 S = 0.572) and BDNF with 11-OH-THC, COOH-THC, COOH-THC-glucuronide, and 7-OH-CBD levels (*p* = 0.005, S = 0.367; *p* = 0.023, S = 0.307; *p* = 0.001, S = 0.447).

### 3.3. Genetics

Single-nucleotide polymorphisms (SNPs) were found to impact cannabinoid concentrations (Table 6).

**Figure 2:**
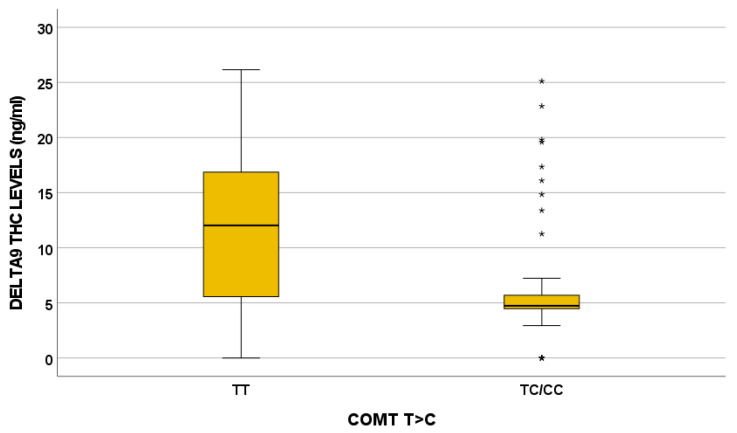
Influence of *COMT* 680 T>C on Δ9-THC plasma levels (ng/mL) (*p* = 0.017). TT N = 11; TC/CC N = 47. Extreme outliers are represented by little stars.

In particular, the *COMT* 680 TC/CC variant influences Δ9-THC, OH-THC, COOH-THC, COOH-THC-glucuronide, and 7-OH-CBD plasma levels. For instance, the effect of the *COMT* 680 T>C variant on Δ9-THC plasma exposure is depicted in Figure 2.

## 4. Discussion

As reported in a previous study by our group, statistically significant differences in cannabinoid plasma exposure between inhaled and oral administration were found in patients with chronic pain treated with MC [35]. In light of this, one of the aims of the present study was to investigate the influence of cannabinoid plasma levels on inflammation-related and neurological markers in individuals treated with MC.

Plasma levels of IL-6, IL-10, and TNF-α were measured as markers of inflammation. Among them, IL-10 levels significantly differed between patients treated with oral and inhaled MC (*p* = 0.009), with lower IL-10 levels in patients using inhaled MC. Cannabinoids play a role in modulating cytokine production within immune cells, since they express the cannabinoid receptor [36]. Tan et al. [37] highlighted that both CBD and THC can suppress the extracellular release of pro- and anti-inflammatory cytokines in an in vitro PBMC model. Notably, treatment with 50 μM cannabidiol significantly reduces the secretion of IL-6 and IL-10. Consequently, higher cannabinoid concentrations, mostly reached via inhalation, are probably related to increased cytokine production.

Furthermore, a statistically significant difference in BDNF levels was observed between patients treated with oral and inhaled MC (*p* = 0.004), with higher levels detected in the latter group. A correlation between BDNF levels and some MC metabolites (11-OH-THC, COOH-THC, COOH-THC-glucuronide, and 7-OH-CBD) was highlighted, suggesting a protective role of cannabinoids. Indeed, BDNF levels in patients treated with MC (median value 1672.6 pg/mL) were higher than BDNF levels in both healthy subjects (167.1 ± 171.2 pg/mL) and in patients with fibromyalgia (113.8 ± 149.6 pg/mL), as reported in the literature [38]. BDNF is involved in neuron growth, development, and survival, both in the central and peripheral nervous system [39], playing a crucial role in synaptic plasticity and neurogenesis [40]. Reduced levels of neurotrophic factors (e.g., BDNF) were described in neurodegenerative disorders [41,42], such as Parkinson’s disease [43], Alzheimer’s disease [44,45], and multiple sclerosis [46,47]. In the literature, a decrease in BDNF plasma levels is associated with impaired brain health [48,49], whereas high levels of BDNF are associated with an improved clinical outcome in patients with schizophrenia [50,51]. THC can upregulate BDNF expression [52,53,54,55]: in fact, in our study, we found that cannabinoids may exert a significant effect on BDNF levels, especially when administered with inhalation. The upregulation of BDNF by THC could be a key mechanism underlying the neuroprotective and neuroplastic effects observed in our patients.

Regarding genetic analysis, SNPs encoding enzymes and transporters associated with cannabinoid metabolism and elimination were explored. In the literature, genetic studies suggested that CYP2C9*2 and *3 genotype frequencies in Caucasians are respectively >18% and 15–20% [29,56]: these gene variants are associated with slower metabolism of THC compared to the CYP2C9*1 variant [29]. In fact, individuals carrying mutations have higher THC concentrations than those with normal alleles (200–300%) [56]. However, no significant association between CYP2C9 polymorphisms and cannabinoid plasma levels was observed in this cohort, consistent with the findings of Papastergiou et al. [57].

Other enzymes relevant to cannabinoid metabolism include CYP3A4 and CYP2C19: the first is implicated in THC and CBD metabolism, while CYP2C19 has an important role in the conversion of CBD in its active metabolite 7-OH-CBD [58], whose formation was positively correlated with enzyme activity but not with *CYP2C19* genotype [58]. In the literature, no association between 7-OH-CBD levels and *CYP2C19* genotype was suggested [58], as reported in our results.

CYP2D6, a key enzyme in the metabolism of psychotropic agents, such as antipsychotics, antidepressants, and anticonvulsants, is also expressed in the brain. Few works investigated the associations between *CYP2D6* and cannabinoids. In our analysis, the *CYP2D6* 840 CG/GG genotype was associated with lower THCA plasma concentrations compared to the CC genotype.

Regarding drug transporters, P-glycoprotein is an efflux protein belonging to the ATP-binding cassette subfamily B member 1, encoded by the *ABCB1* gene [59].

*ABCB1* polymorphisms were studied for their role in cannabis addiction [60,61]: the *ABCB1* 3435 SNP was related to cannabis dependence [29,62]. In a study focused on cannabinoid blood levels, *ABCB1* 3435 T allele carriers had lower THC plasma concentrations than non-T carriers. However, the exact mechanisms were not clarified [63].

*COMT* encodes for catechol-O-methyltransferase [64] and plays a critical role in dopamine metabolism. In the literature, COMT impacts MC response, psychosis risk, and cognitive impairment [29,61]. The role of the *COMT* Val158Met genotype in modulating THC effect on cognition and psychosis was described: high activity associated with the GG genotype (Val/Val) was related with more sensitivity to THC-induced memory impairments compared to the Met allele [64]. *COMT* 680 T>C influences all the cannabinoids quantified in the present study, except for CBD, CBDA, and THCA: in particular, Δ9-THC plasma levels are higher in the TT genotype and lower in patients with the TC/CC genotype (*p* = 0.017).

This study has severe limitations: mainly the small sample size and the imbalance in the number of patients treated with oral versus inhaled MC, which may affect the broader relevance of the results. Nevertheless, as a preliminary investigation, these constraints are understandable and underscore the need for larger, more homogeneous cohorts in future studies. In addition, patient heterogeneity in terms of underlying pathology and MC dosage should be considered when interpreting the findings. Moreover, different pathologies and drug dosages were considered in the study.

## 5. Conclusions

In conclusion, this study suggests a potential impact of some genetic variants on cannabinoid plasma exposure. In addition, for the first time, a possible association between MC treatment, inflammation, and neuronal-related factors was suggested: patients treated with inhaled MC, showing higher cannabinoid concentrations, had lower IL-10 levels and higher BDNF levels. These data may help clarify the protective role of cannabinoids. Further research is needed to confirm the influence of genetic variants on cannabinoid metabolism and to explore the newly reported associations with inflammation and neuroplasticity biomarkers.

## Figures and Tables

**Table 1:** Characteristics of enrolled patients. Abbreviations: IQR = interquartile range (reproduced or adapted from [35], with permission from publisher Alessandra Manca, 2024).

Characteristics	
No. of patients	58
Cigarette smokers, n (%)	19 (32.8%)
Gender (male), n (%)	20 (34.5%)
Caucasian, n (%)	100%
BMI (kg/m^2^), median (IQR)	20.6 (17.9; 23.4)
Age (years), median (IQR)	61 (52; 67)
Fibromyalgia, n (%)	27 (46.6%)
Headache, n (%)	5 (8.6%)
Cancer, n (%)	4 (6.9%)
Polytraumatized patients, n (%)	38 (65.5%)

**Table 2:** *Cannabis* dosages (mg).

*Cannabis* mg	Number of Patients
0–250 mg	25 (43.0%)
300–500 mg	23 (39.7%)
>500 mg	10 (17.3%)

**Table 3:** Differences in median and IQR of plasma cannabinoid in patients treated with inhaled and oral (decoction) cannabis with high levels of THC. IQR = interquartile range (reproduced or adapted from [35], with permission from publisher Alessandra Manca, 2024).

Medical Cannabis with High Levels of THC			
Cannabinoids	Inhaled Cannabis, ng/mL Median (IQR)	Oral Cannabis (Decoction), ng/mL Median (IQR)	*p*-Value
Δ9-THC	14.26 (5.70; 23.99)	5.08 (4.53; 11.04)	0.011
11-OH-THC	0 (0; 11.34)	0 (0; 0)	0.017
COOH-THC	62.99 (27.85; 248.33)	10.53 (6.62; 23.59)	0.004
COOH-THC-glucuronide	511.35 (103.44; 1076.27)	47.92 (7.32; 80.01)	0.003
CBD	5.26 (1.45; 11.45)	2.94 (0.56; 5.73)	0.364
7-OH-CBD	2.26 (0.79; 9.82)	0 (0; 0)	<0.001
THCA	0 (0; 2.11)	3.35 (0; 11.75)	0.127
CBDA	0 (0; 0.41)	0 (0; 0.95)	0.546

**Table 4:** Differences in median and IQR of plasma cannabinoid in patients treated with inhaled and oral (decoction) cannabis with THC and CBD Level Standardized at Concentration of 6.5% and 8%. IQR = interquartile range(reproduced or adapted from [35], with permission from publisher Alessandra Manca, 2024.).

Medical Cannabis with THC and CBD Level Standardized at Concentration of 6.5% and 8%			
Cannabinoids	Inhaled Cannabis, ng/mL Median (IQR)	Oral Cannabis (Decoction), ng/mL Median (IQR)	*p*-Value
Δ9-THC	5.85 (4.60; /)	4.52 (4.18; 5.48)	0.326
11-OH-THC	0 (0; 0)	0 (0; 1.39)	0.412
COOH-THC	43.76 (5.21; /)	11.43 (4.91; 21.70)	0.517
COOH-THC-glucuronide	197.70 (17.81; /)	35.07 (10.35; 63.88)	0.404
CBD	7.83 (3.44; /)	2.12 (0; 3.72)	0.104
7-OH-CBD	0.96 (0; /)	0 (0; 1.67)	0.667
THCA	0 (0; 0)	4.89 (0; 9.04)	0.100
CBDA	0 (0; 0)	1.05 (0; 5.76)	0.118

**Table 5:** Median biomarker levels in plasma for all the analyzed patients.

Biomarkers	Median (Interquartile Range)
Tumor necrosis factor alpha, (ng/mL)	110.40 (98.43; 140.90)
Interleukin-6, (ng/mL)	73.8 (68.1; 88.4)
Interleukin-10, (pg/mL)	245.3 (222.7; 307.3)
Brain-derived neurotrophic factor, (pg/mL)	1672.6 (912.4; 5384.5)
Neurofilament Light Chain, (pg/mL)	6.96 (4.53; 9.72)

**Table 6:** Single-nucleotide polymorphisms influence cannabinoid levels (*p*-values are reported).

Cannabis Metabolites								
	Δ9-THC	OH-THC	COOH-THC	COOH-THC-Glucuronide	CBD	7-OH-CBD	THCA	CBDA
*CYP2D6* 4180 CG/GG							0.023	
*CYP1A1* 2794 AA		0.045						0.020
*COMT* 680 TC/CC	0.017	0.031	0.019	0.031		0.035		
*BSEP* TC/CC					0.037			
*BSEP* CC								0.047
*ABCB1* 1236 CT/TT						0.040		
*CYP1A2* 890 CT/TT	0.033		0.033					

Abbreviations: Δ9-THC = Δ9-tetrahydrocannabinol; CBD = cannabidiol; THCA = tetrahydrocannabinolic acid; CBDA = cannabidiolic acid.

## Data Availability

Data is contained within the article.

## Supplementary Materials

The following supporting information can be downloaded at https://www.mdpi.com/article/10.3390/jox15030066/s1, Table S1: Concomitant class of drugs administered in enrolled patients (reproduced or adapted from [35], with permission from publisher Alessandra Manca, 2024).

## Abbreviations

The following abbreviations are used in this manuscript: 5-HT1A: serotonin 1A receptor; 11-OH-THC: 11-Hydroxy-Δ9-tetrahydrocannabinol; Δ8-THC: Δ8-tetrahydrocannabinol; Δ9-THC: Δ9-tetrahydrocannabinol; BDNF: brain-derived neurotropic factor; CB_1_: Cannabinoid-receptor type 1; CB_2_: Cannabinoid-receptor type 2; CBD: cannabidiol; CBDA: cannabidiolic acid; CBN: cannabinol; CBRs: cannabinoid receptors; CNS: central nervous system; CYP: cytochrome; GPCRs: G-protein-coupled receptors; IL: interleukin; PD: pharmacodynamic; PK: pharmacokinetics; PNS: peripheral nervous system; PPAR: peroxisome proliferator-activated receptors; SNPs: single-nucleotide polymorphisms; TDM: therapeutic drug monitoring; THCA: tetrahydrocannabinolic acid; THC-COOH-: 11-Nor-9carboxy-Δ9-tetrahydrocannabinol; TNF-α: tumor necrosis factor α; TRPV-1: transient receptor potential vanilloid receptor; UHPLC-MS/MS: ultra-high-performance liquid chromatography coupled with tandem mass spectrometry.
