# Supplementary material for: COMT Genetic Variants and BDNF Level Associations with Cannabinoid Plasma Exposure: A Preliminary Study
Source: J Xenobiot. 2025 May 7;15(3):66. doi: 10.3390/jox15030066 (PMC12101174; doi:10.3390/jox15030066)
Supplement: Supplementary file 1 [file jox-15-00066-s001.zip › jox-3515309-supplementary.pdf]

Supplementary Materials: Supplementary table S1

Supplementary table S1. Concomitant class of drugs administered to enrolled patients.

Drugs	*Number of patients (%)*
Antidepressant, n (%)	20 (39.2%)
Anti-inflammatory drugs, n (%)	16 (31.4%)
Opioids, n (%)	21 (41.2%)
Anticonvulsant for neuropathic pain, n (%)	16 (31.4%)
Cardiovascular system drugs, n (%)	15 (29.4%)
Vitamin D supplementation, n (%)	9 (17.6%)
Anti-anxiety medications, n (%)	17 (33.3%)
Other, n (%)	26 (89.7%)
